# MAELASviewer: An Online Tool to Visualize Magnetostriction

**DOI:** 10.3390/s20226436

**Published:** 2020-11-11

**Authors:** Pablo Nieves, Sergiu Arapan, Andrzej Piotr Kądzielawa, Dominik Legut

**Affiliations:** IT4Innovations, VŠB—Technical University of Ostrava, 17. listopadu 2172/15, 70800 Ostrava-Poruba, Czech Republic; sergiu.arapan@vsb.cz (S.A.); andrzej.piotr.kadzielawa@vsb.cz (A.P.K.); dominik.legut@vsb.cz (D.L.)

**Keywords:** magnetostriction, magnetoelastic effects, graphical user interface, web application, visualization

## Abstract

The design of new materials for technological applications is increasingly being assisted by online computational tools that facilitate the study of their properties. In this work, based on modern web application frameworks, the online app MAELASviewer has been developed to visualize and analyze magnetostriction via a user-friendly interactive graphical interface. The features and technical details of this new tool are described in detail. Among other applications, it could potentially be used for the design of magnetostrictive materials for sensors and actuators.

## 1. Introduction

Magnetostriction is a physical phenomenon in which the process of magnetization induces a change in the shape or dimension of a magnetic material. These materials have the advantage that their magnetostrictive properties do not degrade over time as is the case for some poled piezoelectric materials, in addition to their good performance in terms of strains, forces, energy densities, and coupling coefficients [1]. As a result, magnetostrictive materials are widely used in many technological applications like sensors (torque sensors, motion and position sensors, force and stress sensors) and actuators (sonar transducers, linear motors, rotational motors, and hybrid magnetostrictive/piezoelectric devices) where a high magnetostriction is required [2,3,4]. Conversely, in other applications such as electric transformers, motor shielding, and magnetic recording, it is important to reduce the magnetostriction of the magnetic materials [5] as much as possible. Currently, magnetostrictive materials have some drawbacks such as the size and weight required in some applications, high manufacturing cost, and high non-linear and hysteretic effects. These limitations might be overcome by optimizing the design of the materials and their applications. Thanks to modeling techniques, significant improvements have been made in relation to these problems [1].

The study and design of different properties of materials are being increasingly assisted by online visualization tools. For instance, ELATE (an open-source online application for analysis and visualization) is an online user-friendly interface that has been implemented to analyze second-order elastic stiffness tensors and visualize anisotropic mechanical properties [6]. It makes use of a Python module to generate an HTML web page with embedded Javascript for interactive dynamical plots. A similar approach could also be particularly useful in the case of magnetostrictive materials since magnetostriction can be highly anisotropic. Recently, we have developed the MAELAS code [7,8] to calculate magnetostrictive coefficients by automated first-principles calculations. As a complementary online tool of MAELAS, in this work we present the app MAELASviewer to visualize and analyze magnetostriction. The online application is available at https://maelasviewer.herokuapp.com/, while the open-source Python module is available at GitHub repository https://github.com/pnieves2019/MAELASviewer.

## 2. Overview of Magnetostriction

### 2.1. Theoretical Description

The main magnetostriction effects are the Joule effect (length change along the same direction as the magnetic field), Villari effect (magnetization change due to a mechanical stress applied), Wiedemann effect (twisting of a magnetostrictive cylinder when helical magnetic field is applied to the material), and Matteucci effect (induced helical magnetization by a torsion). In this section, we provide a brief introduction to the equations that describe the field-induced magnetostriction (Joule effect and Wiedemann effect), which are implemented in the app MAELASviewer. A more detailed discussion about this topic can be found in [7,9,10,11,12,13].

#### 2.1.1. Relative Length Change Due to the Joule Effect

**Figure 1:**
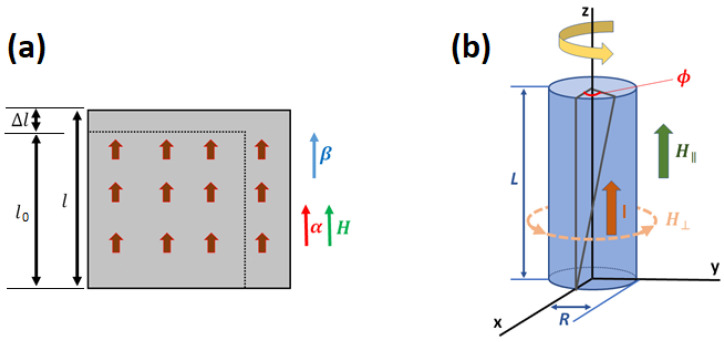
Sketch of the (**a**) Joule effect and (**b**) Wiedemann effect.

Frequently, the physical quantity of interest related to the Joule effect is the change of a material’s length along a measuring direction. It is given by the relative length change $\left(l-l_{0}\right)/l_{0}=\Delta l/l_{0}$, where $l_{0}$ is the initial length of a demagnetized material along a measuring direction β (|β|=1), and *l* is the final length along the same direction β when the system is magnetized along the direction α (|α|=1), see Figure 1. For instance, the relative length change for a cubic single crystal system (point groups 432, $\bar{4}3m$, $m\bar{3}m$) can be written as [9]

(1) $$\begin{array}{cc} \frac{\Delta l}{l_{0}}{|}_{\beta }^{\alpha } & =\lambda^{\alpha }+\frac{3}{2}\lambda_{001}\left(\alpha_{x}^{2}\beta_{x}^{2}+\alpha_{y}^{2}\beta_{y}^{2}+\alpha_{z}^{2}\beta_{z}^{2}-\frac{1}{3}\right)\\ \; & +3\lambda_{111}\left(\alpha_{x}\alpha_{y}\beta_{x}\beta_{y}+\alpha_{y}\alpha_{z}\beta_{y}\beta_{z}+\alpha_{x}\alpha_{z}\beta_{x}\beta_{z}\right), \end{array}$$

where coefficient $\lambda^{\alpha }$ describes the volume magnetostriction (isotropic), while $\lambda_{001}$ and $\lambda_{111}$ are the anisotropic magnetostrictive coefficients that give the fractional length change along the [001] and [111] directions when a demagnetized material is magnetized in these directions, respectively. In the case of hexagonal crystal systems (point groups 6mm, 622, $\bar{6}2m$, 6/mmm), the relative length change reads [9,10,14]

(2) $$\begin{array}{cc} \frac{\Delta l}{l_{0}}{|}_{\beta }^{\alpha } & =\lambda^{\alpha 1,0}\left(\beta_{x}^{2}+\beta_{y}^{2}\right)+\lambda^{\alpha 2,0}\beta_{z}^{2}+\lambda^{\alpha 1,2}\left(\alpha_{z}^{2}-\frac{1}{3}\right)\left(\beta_{x}^{2}+\beta_{y}^{2}\right)\\ \; & +\lambda^{\alpha 2,2}\left(\alpha_{z}^{2}-\frac{1}{3}\right)\beta_{z}^{2}+\lambda^{\gamma ,2}\left[\frac{1}{2}\left(\alpha_{x}^{2}-\alpha_{y}^{2}\right)\left(\beta_{x}^{2}-\beta_{y}^{2}\right)+2\alpha_{x}\alpha_{y}\beta_{x}\beta_{y}\right]\\ \; & +2\lambda^{\epsilon ,2}\left(\alpha_{x}\alpha_{z}\beta_{x}\beta_{z}+\alpha_{y}\alpha_{z}\beta_{y}\beta_{z}\right). \end{array}$$

These magnetostrictive coefficients are related to the normal strain modes for a cylinder [9,10]. In addition to cubic and hexagonal systems, MAELASviewer also supports other single crystals like trigonal systems (point groups 32, 3m, $\bar{3}m$), tetragonal systems (point groups 4mm, 422, $\bar{4}2m$, 4/mmm), and orthorhombic systems (point groups 222, 2mm, mmm). The equation of the relative length change for these systems can be found in [7]. The implemented equations include the magnetostrictive coefficients up to the second-order of the direction cosine polynomial.

Typically, single-crystal magnetostrictive alloys help to understand and characterize the material behavior in the design stage of the material. However, magnetostrictive polycrystalline alloys are more convenient than single crystals for macroscale applications due to the lower production cost and a faster production rate. A widely used approximation to describe magnetostriction for polycrystals is to assume that the stress distribution is uniform through the material. For polycrystalline cubic systems (point groups 432, $\bar{4}3m$, $m\bar{3}m$) the relative change in length may be put into the form [9,15,16]

(3) $$\frac{\Delta l}{l_{0}}{|}_{\beta }^{\alpha }=\frac{3}{2}\lambda_{S}\left[{\left(\alpha \cdot \beta \right)}^{2}-\frac{1}{3}\right]=\frac{3}{2}\lambda_{S}\left[\cos^{2}\xi -\frac{1}{3}\right],$$

where ξ is the angle between magnetization and measuring directions, and

(4) $$\lambda_{S}=\frac{2}{5}\lambda_{001}+\frac{3}{5}\lambda_{111}.$$

This equation describes an isotropic magnetostriction because the relative change in length depends on the magnetization direction in the same way for any measuring direction. Note that polycrystalline cubic systems might not exhibit an isotropic magnetostriction when $\lambda_{001}$ and $\lambda_{111}$ do not have the same sign and order of magnitude, the elastic tensor is not close to being isotropic, and there is a crystallographic texture [12]. On the other hand, the relative change in length for intrinsically isotropic materials like amorphous alloys without growth anisotropy can be well described in the form of Equation (3) [12]. We have also implemented Equation (3) in MAELASviewer.

#### 2.1.2. Deformation Due to the Joule Effect

The total deformation induced by the Joule effect can be described in terms of the displacement vector $u\left(r\right)=r^{\prime }-r$ that gives the displacement of a point at the initial position r in the demagnetized material to its final position $r^{\prime }$ in the magnetized state. For small deformations, the displacement vector can be calculated by solving [17]

(5) $$\begin{array}{c} \epsilon_{ij}^{me}=\frac{1}{2}\left(\frac{\partial u_{i}}{\partial r_{j}}+\frac{\partial u_{j}}{\partial r_{i}}\right),\;\;i,j=x,y,z \end{array}$$

where $\epsilon_{ij}^{me}$ is the equilibrium magnetostrictive strain tensor given by the minimization of both the elastic and magnetoelastic energies [9,10]. The equilibrium magnetostrictive strain tensor is linked to the relative length change via [9,10]

(6) $$\frac{\Delta l}{l_{0}}{|}_{\beta }^{\alpha }=\sum_{i,j=x,y,z}\epsilon_{ij}^{me}\beta_{i}\beta_{j}.$$

For instance, the equilibrium magnetostrictive strain tensor for a cubic single crystal (point groups 432, $\bar{4}3m$, $m\bar{3}m$) reads [9,10]

(7) $$\begin{array}{c} \epsilon^{me}=\lambda^{\alpha }\left(\begin{array}{ccc} 1 & 0 & 0\\ 0 & 1 & 0\\ 0 & 0 & 1 \end{array}\right)+\frac{3}{2}\lambda_{001}\left(\begin{array}{ccc} \alpha_{x}^{2}-\frac{1}{3} & 0 & 0\\ 0 & \alpha_{y}^{2}-\frac{1}{3} & 0\\ 0 & 0 & \alpha_{z}^{2}-\frac{1}{3} \end{array}\right)+\frac{3}{2}\lambda_{111}\left(\begin{array}{ccc} 0 & \alpha_{x}\alpha_{y} & \alpha_{x}\alpha_{z}\\ \alpha_{y}\alpha_{x} & 0 & \alpha_{y}\alpha_{z}\\ \alpha_{z}\alpha_{x} & \alpha_{z}\alpha_{y} & 0 \end{array}\right). \end{array}$$

**Figure 2:**
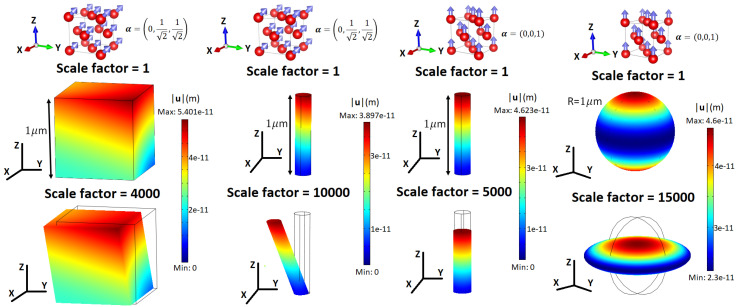
Deformation of single-crystal face-centered cubic (FCC) Ni due to the Joule effect for three material geometries: cube, cylinder, and sphere. The saturated magnetization points to direction $\alpha =(0,1/\sqrt{2},1/\sqrt{2})$ for the cube and cylinder on the left, while it points to α=(0,0,1) for the cylinder and sphere on the right. The color of the surface gives the magnitude of the displacement vector u.

This strain tensor contains three magnetostriction symmetries or normal strain modes for a cube. Namely, a volume change is given by $\lambda^{\alpha }$ that does not depend on magnetization direction, while normal and shear deformations are related to $\lambda_{001}$ and $\lambda_{111}$, respectively [9,10]. In practice, Equation (5) can be solved numerically by finite element modeling (FEM) techniques. To illustrate this method we have used the FEM software COMSOL Multiphysics [18] to calculate the deformation of face-centered cubic (FCC) Ni ($\lambda_{001}=-46\times 10^{-6}$ and $\lambda_{111}=-24\times 10^{-6}$[13]) induced by the Joule effect (without volume magnetostriction $\lambda^{\alpha }=0$) for three simple geometries: cube, cylinder, and sphere with characteristic length 1μ m. The results of the simulation are presented in Figure 2. We see that the magnitude of the maximum displacement vector is of the order $10^{-11}$ m for these material sizes. Increasing the scale factor reveals different types of deformation when magnetization points to $\alpha =(0,1/\sqrt{2},1/\sqrt{2})$ and α=(0,0,1). For α=(0,0,1) we see a normal deformation, while for $\alpha =(0,1/\sqrt{2},1/\sqrt{2})$ there is a combination of normal and shearing deformations due to the contribution of non-diagonal elements ($\epsilon_{yz}$ and $\epsilon_{zy}$) in the magnetostrictive strain tensor Equation (7). A detailed study of magnetostrictive materials with FEM can be found in [19].

#### 2.1.3. The Wiedemann Effect

The helical magnetic field to twist a magnetic rod is achieved by applying a magnetic field along the rod height axis (longitudinal field $H_{\|}$) and an electric current (*I*) through the rod, which induces a circular magnetic field due to Ampère’s circuital law (perpendicular field $H_{⊥}$). In Figure 1 we show a sketch of the Wiedemann effect. For an isotropic magnetic cylindrical rod aligned to the z-axis with height *L* and radius *R*, the twisted angle ϕ induced by a helical magnetic field is [12]

(8) $$\begin{array}{c} \phi \left(z\right)=\frac{3\lambda_{S}Iz}{2AH_{\|}},\;\;0\leq z\leq L \end{array}$$

where $\lambda_{S}$ is the isotropic magnetostrictive coefficient and $A=4\pi R^{2}$ is the area of the cross-section of the rod. The helical field-induced torque can be calculated as [20]

(9) $$\begin{array}{c} \tau =\frac{Y\pi R^{4}\phi \left(L\right)}{4L(1+\sigma )}, \end{array}$$

where *Y* is the Young’s modulus, σ is the Poisson’s ratio, and ϕ(L) is the twisted angle given by Equation (8) evaluated at z=L.

#### 2.1.4. Magnetocrystalline Anisotropy

The magnetocrystalline anisotropy is very important in the design of applications based on magnetostrictive materials since it determines easy and hard magnetic crystallographic directions. The magnetocrystalline anisotropy energy for cubic systems reads

(10) $$\begin{array}{c} E_{a}=K_{1}\left(\alpha_{x}^{2}\alpha_{y}^{2}+\alpha_{x}^{2}\alpha_{z}^{2}+\alpha_{y}^{2}\alpha_{z}^{2}\right)+K_{2}\alpha_{x}^{2}\alpha_{y}^{2}\alpha_{z}^{2}, \end{array}$$

where $K_{1}$ and $K_{2}$ are the magnetocrsytalline anisotropy constants. The corresponding magnetocrystalline anisotropy field is given by

(11) $$\begin{array}{c} \mu_{0}H_{a,i}=-\frac{1}{M_{s}}\frac{\partial E_{a}}{\partial \alpha_{i}}\;\;i=x,y,z \end{array}$$

where $M_{s}$ is the saturation magnetization and $\mu_{0}$ is the vacuum permeability. In our simulation of the Joule effect we include the magnetocrystalline anisotropy field and external magnetic field (H). At the equilibrium the magnetization points to the direction of the effective field

(12) $$\begin{array}{c} H_{eff}=H+H_{a} \end{array}$$

Additionally, MAELASviewer also contains 3D visualization tools for the analysis of the magnetocrystalline anisotropy energy.

### 2.2. Magnetostrictive Materials

The first measurements of magnetostriction were made in Fe, Ni, and their alloys after the discovery of this phenomenon by James P. Joule in 1842 [21]. Despite the low magnetostriction of these itinerant magnets ($\lambda <10^{-4}$), numerous applications were developed in the first half of the 20th century, including telephone receivers, hydrophones, scanning sonar, fog horns, oscillators, and torque sensors [1]. In the second half of the 20th century, giant magnetostrictive materials were discovered in elementary rare-earth (R) metals (under low temperature and high magnetic field) and compounds containing R and transition metals ($\lambda >10^{-3}$). In particular, the highest magnetostrictions were found in the RFe_2_ compounds with a Laves phase C15 structure (face-centered cubic) type [10]. For instance, Terfenol-D (Tb${}_{0.27}$Dy${}_{0.73}$Fe${}_{2}$) is a widely used magnetostrictive material thanks to its giant magnetostriction along the [111] crystallographic direction ($\lambda_{111}=1.6\times 10^{-3}$) under moderate magnetic fields (<160 kA/m) at room-temperature [11]. Beyond cubic systems, the research into magnetostrictive materials has been focused on hexagonal crystals like RCo${}_{5}$ (space group 191), hexagonal and trigonal R${}_{2}$Co${}_{7}$ and R${}_{2}$Co${}_{17}$ series, and tetragonal R${}_{2}$Fe${}_{14}$B [9,22]. More recently, the problem of R availability [23] has also motivated the exploration of R-free magnetostrictive materials like Galfenol (Fe-Ga), spinel ferrites (CoFe${}_{2}$O${}_{4}$), Nitinol (Ni-Ti alloys), Fe-based Invars, and Ni_2_MnGa [1,24,25]. The experimental magnetostrictive coefficients for some representative cubic crystals are shown in Table 1.

### 2.3. Applications of Magnetostriction

**Figure 3:**
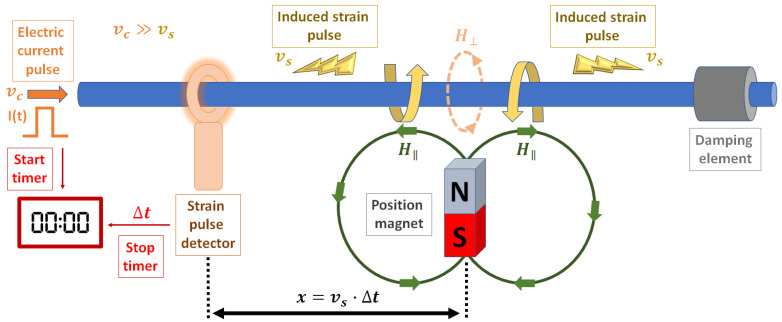
Schematic of a magnetostrictive linear position sensor based on the Wiedemann effect.

The discovery of giant magnetostrictive materials like Terfenol-D has enabled the development of novel applications based on magnetostriction like advanced sensors and actuators. Presently, the most common magnetostrictive sensors are torque sensors (used in passenger cars, tractors, truck and off-highway vehicle powertrains, manufacturing machinery, and stationary power plants) [2,26], motion and position sensors [2] (mainly used in the automotive industry), and force and stress sensors (used in vehicle active suspensions and engine mounts, active vibration control, manufacturing control, monitoring overloads on bridges, and active control of buildings against seismic events) [1,2]. A magnetostrictive linear position sensor based on the Wiedemann effect is depicted in Figure 3 [27]. In this sensor, the position magnet is attached to whatever is being measured and generates the longitudinal field ($H_{\|}$). The transverse field ($H_{⊥}$) is created by a short electric current pulse through the ferromagnetic rod, which starts a timer and induces a strain pulse due to the twist of the rod close to the position magnet (Wiedemann effect). This strain pulse travels at the speed of sound in the rod ($v_{s}$) until is detected by a pickup, stopping the timer. The elapsed time indicated by the timer (Δt) then represents the distance between the position magnet and the pickup ($x=v_{s}\cdot \Delta t$). Concerning actuators, the main applications are sonar transducers [28], linear motors [4,29], rotational motors [30], and hybrid magnetostrictive/piezoelectric devices [31].

## 3. Methodology

### 3.1. Software Details

MAELASviewer was created with Dash, which is a productive Python framework for building web applications [32]. Dash uses Flask as the web framework [33]. Visualization of 3D surfaces is done using the Python open source graphing library Plotly [34]. We also make use of NumPy library for some math operations. The online application is deployed to the cloud platform Heroku [35] that allows users to build, deliver, monitor, and scale apps easily. Alternatively, one can also run MAELASviewer offline by executing the Python module available at the GitHub repository https://github.com/pnieves2019/MAELASviewer.

### 3.2. Modeling of the Joule Effect

#### 3.2.1. Mapping the Joule Effect to a Sphere

**Figure 4:**
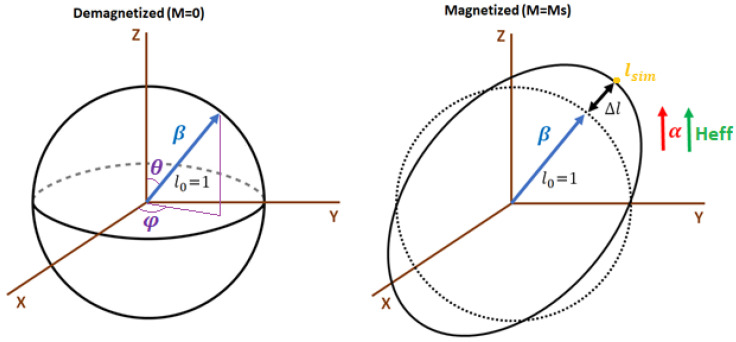
The Joule effect mapped to a sphere. In this model we assume that the magnetization is saturated in the direction of the effective field ($\alpha ∥H_{eff}$). Symbols *M* and $M_{s}$ stand for macroscopic magnetization and saturation magnetization, respectively. The dash line on the right represents the original unit sphere (demagnetized state).

In MAELASviewer we have implemented a tool to map the longitudinal deformation of the Joule effect to a sphere. Namely, the length $l_{0}$ of the material in any arbitrary measuring direction β is represented by a sphere with radius 1 ($l_{0}=1$), so that it does not depend on the geometry of the material. Here, the direction β is written in spherical coordinates β=(sinθcosφ,sinθsinφ,cosθ), where θ and φ are the polar and azimuthal angles, respectively. Figure 4 shows a sketch of the Joule effect mapped to a sphere. In the magnetized state, the distance between a point on the surface of the distorted sphere and the origin (0,0,0) describes the simulated length $l_{sim}$ along direction β.

Magnetostriction is a small effect that is hard to visualize. In order to facilitate the 3D visualization of the distorted sphere in the simulation, we multiply the relative length change by a scale factor *s*, which can be modified by the user. For instance, Equation (1) is implemented as

(13) $$\begin{array}{cc} \frac{\Delta l}{l_{0}}{|}_{\beta }^{\alpha } & =s\cdot [\lambda^{\alpha }+\frac{3}{2}\lambda_{001}\left(\alpha_{x}^{2}\beta_{x}^{2}+\alpha_{y}^{2}\beta_{y}^{2}+\alpha_{z}^{2}\beta_{z}^{2}-\frac{1}{3}\right)\\ \; & +3\lambda_{111}\left(\alpha_{x}\alpha_{y}\beta_{x}\beta_{y}+\alpha_{y}\alpha_{z}\beta_{y}\beta_{z}+\alpha_{x}\alpha_{z}\beta_{x}\beta_{z}\right)]. \end{array}$$

Note that this scaling preserves the ratio between the magnetostrictive coefficients. Obviously, the case with s=1 corresponds to the real situation. The length calculated in the simulation ($l_{sim}$) along to the direction β is

(14) $$\begin{array}{cc} l_{sim}{|}_{\beta }^{\alpha } & =1+s\cdot [\lambda^{\alpha }+\frac{3}{2}\lambda_{001}\left(\alpha_{x}^{2}\beta_{x}^{2}+\alpha_{y}^{2}\beta_{y}^{2}+\alpha_{z}^{2}\beta_{z}^{2}-\frac{1}{3}\right)\\ \; & +3\lambda_{111}\left(\alpha_{x}\alpha_{y}\beta_{x}\beta_{y}+\alpha_{y}\alpha_{z}\beta_{y}\beta_{z}+\alpha_{x}\alpha_{z}\beta_{x}\beta_{z}\right)], \end{array}$$

where we took into account that $l_{0}=1$. The same procedure is applied to the other supported crystal systems. In addition to 3D plots, MAELASviewer generates interactive 2D plots showing the cross-section of the distorted sphere. As a reference, in the 2D plots it also prints the cross-section of the unit sphere representing the demagnetized state. However, in these plots it is hard to distinguish the cross-section of the distorted and undistorted spheres since magnetostriction makes a very small change in the length. Therefore, it is recommended that the scale factor is increased in order to see both curves clearly.

#### 3.2.2. Applications and Physical Interpretation

**Figure 5:**
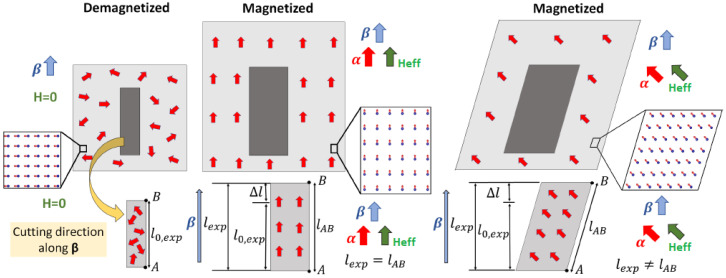
Schematic showing that if we cut the material along the measuring direction β and the effective magnetic field induces a normal deformation, then the length in the direction β, $l_{exp}$, given by Equation (15), is equal to the total length $l_{AB}$ defined as the distance between the new positions of points A and B in the deformed material. However, if the field induces a shearing deformation then $l_{exp}$ is not equal to $l_{AB}$.

Among other possible applications, this procedure can be a useful tool to determine the length change due to the Joule effect in the cutting direction of a crystal. For instance, let us consider a material cut along a crystallographic direction β with length $l_{0,exp}$ in the demagnetized state. The length in the direction β of the material in the magnetized state ($l_{exp}$) can easily be obtained with MAELASviewer by setting the magnetic field, the corresponding values of the magnetostrictive coefficients and magnetocrystalline anisotropy constants, and using the following equation

(15) $$\begin{array}{c} l_{exp}=\frac{l_{0,exp}}{s}\left(s+l_{sim}-1\right), \end{array}$$

where $l_{sim}$ is the simulated length value printed by the interactive app at the surface of the distorted unit sphere along direction β, and *s* is the scale factor set by the user. We see that after setting the scale factor equal to 1 (s=1) then one just needs to multiply $l_{sim}$ by $l_{0,exp}$, that is, $l_{exp}=l_{sim}\cdot l_{0,exp}$. When the user clicks on the surface of the distorted sphere, MAELASviewer prints the components of vector $l_{sim}$ so that it gives both the length $l_{sim}=\left|l_{sim}\right|$, and direction β since it is aligned to $l_{sim}$ ($\beta \|l_{sim}$). To gain more insight into this method, let us cut the material in the measuring direction β and measure the initial length $l_{0,exp}$ in the demagnetized state as the distance between points A and B depicted in Figure 5. If we now apply a magnetic field that induces only a normal deformation, then $l_{exp}$ is equal to the total length $l_{AB}$ defined as the distance between the new positions of points A and B in the deformed material. However, we see that if the magnetic field induces a shearing deformation, then $l_{exp}$ is not equal to $l_{AB}$.

**Figure 6:**
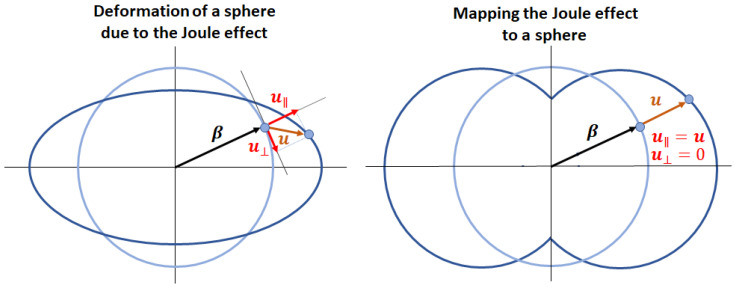
Comparison between the shape deformation of a sphere induced by the Joule effect and the shape generated by mapping the Joule effect to a sphere.

It is important to note that this method gives only the length change in the measuring direction but not the overall real shape deformation of a material induced by the Joule effect, not even for a sphere. In Figure 2, we observe that the real shape deformation of the sphere corresponds to an ellipsoid. This shape is different to the shape of the distorted sphere generated by mapping the Joule effect to a unit sphere that is implemented in MAELASviewer. As we see in Figure 6, the reason for that is the lack of a transverse component of the displacement vector in this procedure ($u_{⊥}=0$, $u_{\|}=u$).

#### 3.2.3. Magnetic Field and Temperature Effects

The current version of MAELASviewer is designed to work in the saturated magnetostriction regime, that is the magnetization is assumed to be saturated along the effective magnetic field ($\alpha ∥H_{eff}$). However, notice that important aspects and applications of magnetostriction also take place below the saturated magnetostriction. In the not saturated regime one can take into account the effects of different magnetic domain orientations on the fractional length change by performing a spatial average over magnetization directions, that is, replacing $\alpha_{i}\alpha_{j}$ by their spatial average $<\alpha_{i}\alpha_{j}>$[12]. In the demagnetized state, we have $<\alpha_{i}^{2}>=1/3$ and $<\alpha_{i}\alpha_{j}>=0$ with i≠j so that the anisotropic fractional length change is zero ($l=l_{0}$). Once the magnetic field is applied, magnetostriction is driven by the domain structure of the material, making the analysis of this process more complicated [12]. The implementation of these features into MAELASviewer, at least to some extent, is an interesting possibility that is worth exploring in the future.

In a remanence state close to saturation magnetization (all magnetic domains are almost aligned in the absence of applied field), magnetic fields lower than the magnetocrystalline anisotropy field ($H<H_{a}$) can also influence the magnetostriction by rotating the magnetization, e.g., when the field is applied perpendicular to magnetization that is parallel to an easy axis ($\alpha \|H_{a}$, α⊥H). In this case, a quadratic dependence on the magnetic field is observed in magnetostriction until the magnetization is completely aligned to the magnetic field (α∥H) [13]. This process can be analyzed with MAELASviewer since it calculates the direction of the equilibrium magnetization by a direct numerical integration of the Landau-Lifshitz-Gilbert equation. The minimum magnetic field ($H_{min}$) required to achieve the alignment of magnetization to certain crystallographic direction is important when designing magnetostrictive materials. Typically, low values of $H_{min}$ are highly desirable to make feasible applications. However, magnetic materials with giant magnetostriction usually exhibit high magnetocrystalline anisotropy that can induce very hard directions. An optimal balance between magnetostriction and magnetocrystalline anisotropy was achieved in Terfenol-D by adding Dy to TbFe${}_{2}$ [11]. Therefore, combining the visualization and analysis of both magnetostriction and magnetocrystalline anisotropy energy could allow identification of crystallographic directions with high magnetostriction to magnetocrystalline anisotropy ratios.

Another important fact to take into account when designing magnetostrictive materials is the temperature, since this greatly influences magnetostriction. Close to the Curie temperature ($T_{C}$), magnetostriction decreases very rapidly with temperature and it becomes zero above $T_{C}$. Hence, temperature limits the range of applicability of magnetostrictive materials, especially in devices working under extreme operating conditions. In the saturated magnetostriction regime, the fractional length change depends on the temperature only via the magnetostrictive coefficients. Therefore, MAELASviewer gives the fractional length change at the temperature that corresponds to the values of the magnetostrictive coefficients used as inputs.

### 3.3. Modeling of the Wiedemann Effect

In the simulation of the Wiedemann effect, we consider an isotropic cylindrical rod as it is described in Section 2.1.3. The twisted angle ϕ and torque τ are calculated via Equations (8) and (9), respectively, using the input parameters provided by the user. In the 3D visualization of the rod, it is also plotted the transverse and longitudinal magnetic fields in the exterior of the rod at z=L/2. The transverse field is calculated by applying the Biot–Savart law for a finite wire [36]. Note that the magnetic field generated by the magnetization of the rod is not plotted by the simulation.

## 4. Graphical User Interface

MAELASviewer has a user-friendly interface with interactive plots that allow the user to visualize the relative length change of a material along an arbitrary direction β as a function of the external magnetic field direction H, magnetostrictive coefficients λ and magnetocrystalline anisotropy constants (Joule effect). For each available crystal system, the simulation shows four interactive figures: (i) a 3D visualization of the orientation of lattice vectors, magnetization, and external magnetic field, (ii) a 3D visualization of the total length *l* and relative length change $\Delta l/l_{0}$, (iii) 2D visualization of the cross-section for relative length change in the XY, XZ, and YZ planes, and (iv) 3D visualization of the magnetocrystalline anisotropy energy. Additionally, it includes tables with the experimental data of magnetostrictive coefficients for some magnetic materials.

**Figure 7:**
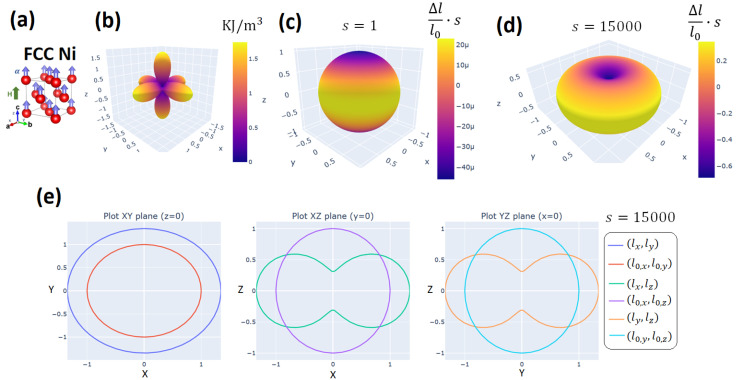
Mapping the Joule effect to a sphere for single-crystal FCC Ni at room-temperature under an external magnetic field parallel to the z-axis. (**a**) Unit cell of FCC Ni. (**b**) 3D visualization of the magnetocrystalline anisotropy energy. 3D visualization of the simulated length $l_{sim}$ via Equation (14) with scale factor (**c**) s=1 and (**d**) s= 15,000. The color of the surface corresponds to the relative length change multiplied by the scale factor. (**e**) Cross-section of the simulated length $l_{sim}$ with scale factor s= 15,000 in the planes XY (z = 0), XZ (y = 0), and YZ (x = 0).

**Figure 8:**
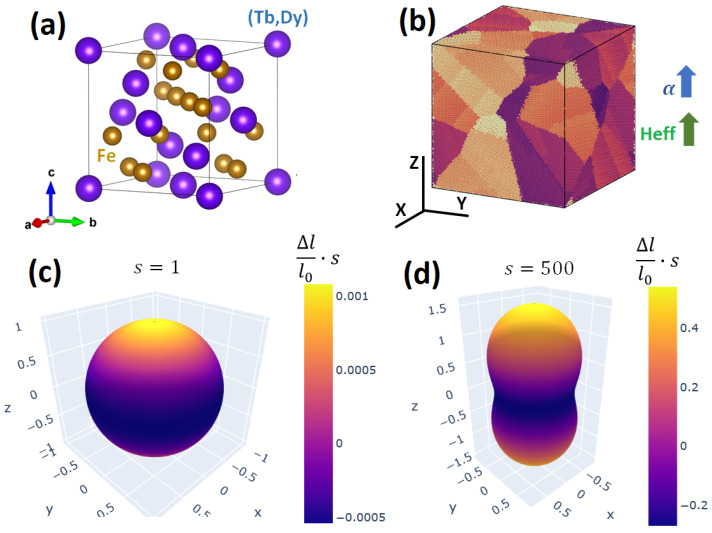
Mapping the Joule effect to a sphere for polycrystalline Terfenol-D at room-temperature under an effective magnetic field parallel to the z-axis. (**a**) Unit cell of Terfenol-D. (**b**) Atomistic model of polycrystalline Terfenol-D. 3D visualization of the simulated length $l_{sim}$ with scale factor (**c**) s=1 and (**d**) s=500. The color of the surface corresponds to the relative length change multiplied by the scale factor.

As an example, we used MAELASviewer to map the Joule effect to a sphere for FCC Ni at room-temperature under a strong external magnetic field parallel to the z-axis ($H\;\gg \;H_{a}$). In the simulation, we set the experimental values for the anisotropic magnetostrictive coefficients $\lambda_{001}=-46\times 10^{-6}$ and $\lambda_{111}=-24\times 10^{-6}$ without volume magnetostriction ($\lambda^{\alpha }=0$), and magnetocrystalline anisotropy constants ($K_{1}=-4.5$ KJ/m^3^, $K_{1}=-2.3$ KJ/m^3^ [13]. The figures generated by MAELASviewer are depicted in Figure 7. We see that the external magnetic field along the z-axis induced a compression along the z-axis due to the negative value of $\lambda_{001}$. This effect can clearly be seen using the scale factor s= 15,000. Note that this shape does not correspond to the deformation induced by the Joule effect in a sphere, see Section 2.1.2. In Figure 7b, we clearly see the easy and hard magnetic directions along [1,1,1] and [1,0,0], respectively. As an example for polycrystals, in Figure 8 we mapped the Joule effect to a sphere for polycrystalline Terfenol-D at room-temperature under an effective magnetic field parallel to the z-axis setting $\lambda_{s}=1080\times 10^{-6}$[1]. We observed a giant expansion of the material along the magnetic field that is mainly originated by the grains with crystallographic axis [1,1,1] nearly oriented along the magnetic field ($\lambda_{111}\gg \lambda_{001}$). This extraordinary magnetostrictive response makes it very useful for many technological applications.

**Figure 9:**
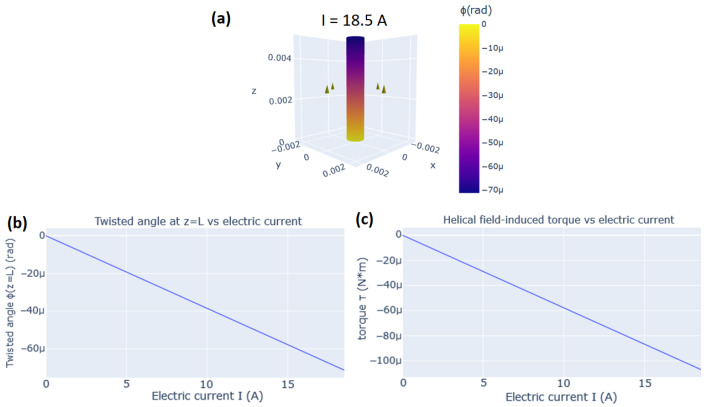
Simulation of the Wiedemann effect for a Ni wire. (**a**) 3D visualization of the twisted angle as function of the z-coordinate, ϕ(z), of the rod with applied electric current I=18.5 A. The green arrows at z=L/2 represent the magnetic field $H_{\|}+H_{⊥}$. (**b**) Twisted angle at z=L, ϕ(L), versus the electric current *I*. (**c**) The helical field-induced torque τ versus the electric current *I*.

To illustrate the visualization of the Wiedemann effect in MAELASviewer, we simulated the experiment performed in 1919 by Pidgeon on annealed Ni wire with radius R=0.5 mm under a longitudinal magnetic field $H_{\|}=22.3$ kA/m [12,37]. Here, we used the experimental value for the isotropic magnetostrictive coefficient $\lambda_{s}=-36\times 10^{-6}$[12], and we set L=5 mm for the rod height. The applied electric current ranged from $I_{min}=0$ A to $I_{max}=18.5$ A. Additionally, we used the experimental values of Ni for the Young’s modulus Y=200 GPa and Poisson’s ratio σ=0.31 to calculate the helical field-induced torque τ. The results generated by MAELASviewer are shown in Figure 9. The 3D visualization of the twisted angle as function of z-coordinate ϕ(z) of the rod is plotted for the maximum applied electric current $I_{max}=18.5$ A. This yields a twisted angle at z=L equal to ϕ(L)=−71.3μrad. MAELASviewer also plots the twisted angle at z=L and induced torque for the all selected range of applied electric current. This simulation reproduces the experimental twisted angle at z=L given by $\phi \left(L\right)=[-6.74\times 10^{-5}-2.4\times 10^{-9}\left(I/A\right)]L$ rad, where $A=4\pi R^{2}$ is the area of the cross-section of the cylindrical rod [12].

## 5. Conclusions

Based on recent advancements in the software for online interactive applications, we have developed the app MAELASviewer to visualize and analyze magnetostriction. Thanks to its user-friendly interface, it allows users to easily simulate how an external magnetic field changes the size of a magnetic material for different crystal systems. Among other possible applications, this tool can be used to determine the length change due to the Joule effect along the cutting direction of a crystal. Therefore, it might be helpful in the design and manufacturing stages of a magnetostrictive material for some applications based on the Joule or Wiedemann effects, like sensors and actuators. Additionally, from a fundamental point of view, it allows users to gain an accurate and deep intuitive understanding of the anisotropic magnetostrictive responses of magnetic materials. It could also be used as a complementary tool of MAELAS code to further analyze the magnetostrictive coefficients calculated by automated first-principles calculations.

## Figures and Tables

**Table 1:** Magnetostrictive coefficients of some cubic crystals.

Material	Temperature (K)	$\lambda_{001}\left(\times 10^{-6}\right)$	$\lambda_{111}\left(\times 10^{-6}\right)$	$\lambda_{s}\left(\times 10^{-6}\right)$
(BCC) Fe	4.2	26 ${}^{a}$	−30 ${}^{a}$	-
(BCC) Fe	300	21 ${}^{a}$	−21 ${}^{a}$	−7 ${}^{a}$
(FCC) Ni	4.2	−60 ${}^{a}$	−35 ${}^{a}$	-
(FCC) Ni	300	−46 ${}^{a}$	−24 ${}^{a}$	−34 ${}^{a}$
(Laves phase C15) SmFe_2_	4.2	30 ${}^{b}$	−4100 ${}^{b}$	-
(Laves phase C15) DyFe_2_	4.2	−70 ${}^{b}$	3000 ${}^{b}$	-
(Laves phase C15) TbCo${}_{2}$	4.2	−1200 ${}^{b}$	4500 ${}^{b}$	-
(Laves phase C15) ErCo${}_{2}$	4.2	−1000 ${}^{b}$	−2500 ${}^{b}$	-
(Laves phase C15) Terfenol-D	300	90 ${}^{c}$	1600 ${}^{c}$	1080 ${}^{c}$

${}^{a}$ Ref. [13], ${}^{b}$ ref. [22], ${}^{c}$ ref. [1].
